# Association of Serum Bisphenol A with Hypertension in Thai Population

**DOI:** 10.1155/2015/594189

**Published:** 2015-02-16

**Authors:** Wichai Aekplakorn, La-or Chailurkit, Boonsong Ongphiphadhanakul

**Affiliations:** ^1^Department of Community Medicine, Faculty of Medicine, Ramathibodi Hospital, Mahidol University, Bangkok 10400, Thailand; ^2^Department of Medicine, Faculty of Medicine, Ramathibodi Hospital, Mahidol University, Bangkok 10400, Thailand

## Abstract

*Objective*. The present study aimed to examine the association between serum BPA and hypertension and evaluated whether it was influenced by estradiol level. *Methods*. A subsample of 2588 sera randomly selected from the Thai National Health Examination Survey IV, 2009, was measured for serum BPA and estradiol. Logistic regression was used to examine the association controlling for age, sex, diabetes, body mass index, and estradiol level. *Results*. Compared with the lowest quartile, the adjusted odds ratio (AOR) of hypertension for the fourth quartile of serum BPA was 2.16 (95% CI 1.31, 3.56) in women and 1.44 (0.99, 2.09) in men. There was no interaction between serum BPA and estradiol level. For analysis using log(BPA) as a continuous variable, the AOR per unit change in log(BPA) was 1.09 (95% CI 1.02, 1.16). Among postmenopausal women, the AOR for the fourth quartile of BPA was 2.33 (95% CI 1.31, 4.15) and, for premenopausal women, it was 2.12 (95% CI 0.87, 5.19). *Conclusion*. Serum BPA was independently associated with hypertension in women and was not likely to be affected by estrogen; however, its mechanism related to blood pressure needs further investigation.

## 1. Introduction

Bisphenol A (BPA) is a widely used chemical in the production of plastic food containers in many consumer products and of epoxy resins in dental fillings. Exposure to BPA is very common in almost all US adults [1], Asian population [2], and about 80% of Thai population [3]. Recently, this chemical has received much attention for its association with many health impacts [4]. BPA, a xenoestrogen, might mimic estrogen and disrupt the function of estrogen through estrogen receptor [5]. So far, health effects associated with exposure to BPA as reported in the literature include estrogenic activity, thyroid hormone disruption [6], pancreatic beta cell function disturbance, and increased risk of diabetes [3]. Epidemiological studies have shown that BPA in urine and serum is associated with diabetes [7–11], obesity [12], and self-reported cardiovascular diseases [13–15]. Bae et al. reported an association of urinary BPA with hypertension, especially among those not previously known to have hypertension [16]. Shankar and Teppala reported the association of urinary BPA with hypertension independent of other risk factors [17].

Globally, high blood pressure is the most common risk factor for cardiovascular diseases death. It contributed to 40% of cardiovascular deaths in 2010 [18]. Although conventional risk factors for hypertension such as salt intake and obesity were described [19], knowledge of other environmental risk factors and their role in hypertension might be useful in prevention and control of the condition. As estrogen has vasorelaxation effect [20, 21], it is not clear whether exposure to BPA is associated with high blood pressure and in what direction. Previous study has reported an association between urinary BPA and hypertension; however, there has been no prior study using serum BPA as exposure indicator. It is less clear whether high BP is related to BPA and the effect is influenced by estrogen level. The objective of the present study was to determine the association between serum BPA and blood pressure and if so whether the effect was related to estrogen level or menopausal status.

## 2. Methods

Data were from Thai National Health Examination Survey (NHES) 2009. Details of sampling methods were described previously elsewhere [22]. In brief, a subsample of 2588 sera from the NHES IV was randomly selected and measured for the level of bisphenol A and estradiol. The sample was randomly selected according to sex and 6 age groups (15–29, 30–44, 45–59, 60–69, 70–79, and 80–89). 25 samples were selected from each stratum, and a total of 2588 samples were available.

The NHES was approved by the Ethics Committee of Ministry of Public Health, and written informed consent was obtained from all participants. This study was also approved by the Ethics Committee of Ramathibodi Hospital, Faculty of Medicine, Mahidol University.

Demographic data such as age and sex were included. Body mass index (BMI) was calculated as weight in kilograms divided by the square of height in meters. Serum BPA levels were measured by competitive ELISA (IBL International GmH, Hamburg, German) with an intra-assay and interassay precision of 11.8% and 13.6%, respectively. Serum levels of 17*β*-estradiol (E2) were determined by electrochemiluminescence immunoassay on a Cobase 411 analyzer (Roche Diagnostics GmbH). The intra-assay precision was 4.3%.

Measurements of blood pressure were performed by trained field research assistants using automatic sphygmomanometer Microlife model A100 (Microlife AG, Widnau, Switzerland) with appropriate arm cuffs. Prior to measurement, participants rested for 5 min in a sitting position according to the standard protocol [23]. Three measurements of blood pressure were obtained from each participant. An average of the second and third values was calculated and used in the analysis.

Alcohol drinking is defined as self-reported alcohol beverage consumption in the past 12 months. Smoking is defined as follows: current cigarette smokers were defined as individuals who had smoked ≥100 cigarettes during their lifetime and at the time of interview responded that they smoked every day or some days [24].

### 2.1. Definition of Hypertension

Hypertension is defined as mean systolic blood pressure (SBP) equal to or greater than 140 mmHg or mean diastolic blood pressure (DBP) equal to or greater than 90 mmHg or on medication to lower blood pressure in the past two weeks. Unaware hypertension is defined as individuals with hypertension, who have never been told by health professionals that they had hypertension. In the analysis, we also examined the association with those who treated hypertension in the past two weeks and for those who were diagnosed but did not receive medication to lower their blood pressure.

### 2.2. Statistical Analysis

All analyses took into account the complex survey design by weighting for the total population. Descriptive statistical analyses of percentages, means, and standard variation (SD) were performed. Student's unpaired *t*-test was used to compare means between groups. Geometric mean of BPA was calculated and used due to the skewness of BPA distribution. BPA level was categorized into quartile. Means and proportions of several clinical variables including pulse pressure, SBP, DBP, fasting plasma glucose, serum estrogen level, diabetes, and several hypertension status variables were calculated according to quartile of BPA and then compared. Logistic regression was used to examine the association between BPA and blood pressure and hypertension status. First, each hypertension status variable was regressed on the log(BPA) as a continuous variable in the model. Potential confounding variables that were adjusted included age, sex, BMI, alcohol drinking, smoking and diabetes status, and estradiol level. We tested for the interaction between BPA and estrogen level by adding an interaction term between log(BPA) and estradiol level in the model. To handle the problem of missing values of log(BPA) for undetected BPA level as zero, individuals with zero BPA level were assigned to 0.005 (an average of the lowest value and zero). We also ran logistic regression using quartile of BPA as categorical variable in the model. To determine whether the effects of BPA or hypertension status varied by menopausal status, an additional stratified analysis for the association of BPA and hypertension was performed separately for premenopausal and postmenopausal status. There were 19 postmenopausal women having history of taking hormone supplement; however, excluding this group of women did not change the results, so we did not exclude them from the analysis. All the analyses were performed using Stata software version 10.1 (StataCorp. College Station, Texas, USA). Statistical significance was set at a *P* value of <0.05.

## 3. Results

Table 1 gives the basic characteristics of the 2,558 participants. Mean age of the participants was 40.37 (SD 16.81) years. Overall geometric mean of BPA was 0.34 (SD 0.02) ng/mL with a slightly higher value but was not significant in men compared to that in women. Prevalence of hypertension in men and women was relatively similar at 18%. The pronounced differences were the significantly higher prevalence of smoking and alcohol drinking in men compared to women. Mean of estradiol level in women was significantly higher than that in men (*P* < 0.001). Women had a higher BMI compared with men.

Table 2 shows the weighted geometric mean BPA by sex and several clinical conditions. Mean BPA was significantly higher in individuals with hypertension than in those with normotension (*P* < 0.01), especially in women (*P* < 0.01). Geometric means of BPA were also higher among other hypertension variables designated for high blood pressure such as those with unaware hypertension and among those with high SBP ≥ 140 mmHg. There were no significant differences in BPA levels between smokers and nonsmokers as well as between alcohol drinkers and nondrinkers.

**Figure 1:**
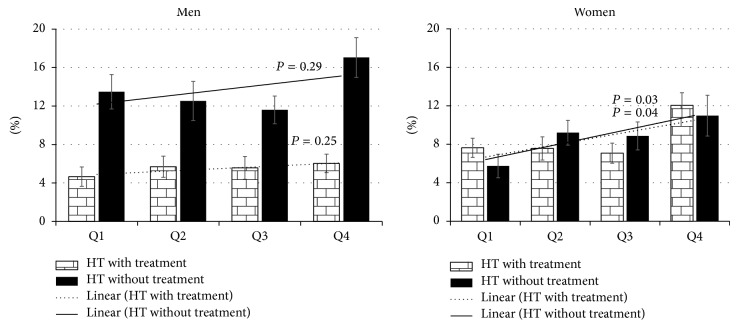
Age-adjusted percentages of hypertension with and without treatment by quartile of BPA level in men and women.

Table 3 shows the distribution of several blood pressure variables as varied by quartiles of BPA. Overall, SBP and DBP were not significantly varied by BPA quartiles. Compared with the lowest quartile, prevalence of all hypertension, unaware hypertension, and those treated or not treated were significantly highest in the fourth quartile. To explore whether the association of BPA with hypertension was related to level of estrogen or menopausal status, an interaction term was added in the logistic regression models.  Figure 1 shows the age-adjusted percentages of hypertension with and without treatment according to quartile of BPA level in men and women. Compared to the first quartile, the age-adjusted hypertension prevalence significantly increased in the fourth quartile for women for both hypertension with treatment and hypertension without treatment (*P* = 0.03 and 0.04, resp.), but not in men.

Table 4 gives odds ratios of association between log(BPA) and hypertension status controlling for age, BMI, diabetes, smoking, and estradiol level among females with an interaction between log(BPA) and estradiol level. Log(BPA) was significantly associated with hypertension with an odds ratio of 1.09 (1.02, 1.16). Using several definitions for hypertension status (unaware hypertension (HT), SBP ≥ 140 mmHg, DBP ≥ 90 mmHg, treated HT, or untreated HT) yielded similar results of no interaction between BPA and estradiol.  Table 5 gives the association between BPA quartile and a number of different definitions of hypertension. Overall, compared with the first quartile, those in the highest quartile of BPA had a significant 76% higher chance of hypertension. The association appeared to be stronger among women with an adjusted odds ratio of 2.16 (95% CI 1.31, 3.56) for the highest quartile. The association was even slightly stronger for unaware hypertension (adjusted OR 2.54, 95% CI 1.11, 6.83). We further performed a stratified analysis according to menopausal status.  Table 6 shows the significant associations of hypertension status with the highest quartile of BPA among postmenopausal women, but not among premenopausal women (2.33, 95% CI 1.31, 4.15, and 2.12, 95% CI 0.87, 5.19, resp.). An additional analysis using log(BPA) as continuous variable as shown in Table 7 yielded consistent results of significant associations with hypertension status with AORs ranging from 1.02 to 1.15.

## 4. Discussion

The present study revealed that serum BPA was independently associated with hypertension. The positive association was found for all hypertension and unaware HT among women. The association was not likely to be confounded by age, sex, BMI, diabetes, and estrogen level. Although there was no clear interaction between BPA and estrogen level, the association between BPA and hypertension appeared to be stronger among postmenopausal women. The positive association between serum BPA and hypertension was consistent with studies using urinary BPA as surrogate. Bae and colleagues reported that urinary BPA was positively associated with blood pressure but negatively associated with heart rate variability [16]. Shankar and colleague analyzed data from NHANES 2003-2004 and found the association of urinary BPA with hypertension [17].

Mechanism of action of BPA on cardiovascular system is complex and the mode of action on blood pressure is unclear; however, there are several pathways that might be possible. A study suggests that exposure to BPA is related to insulin resistance and its role in weight gain and obesity [25]. Activation of ER*β* can reduce systematic arterial pressure via autonomic influence [26]. Hypertension and vascular dysfunction have been shown in whole body ER*β* knockout mice [27, 28]. BPA is associated with decrease in free thyroid hormone [6], and hypothyroid has been reported to be associated with hypertension [29]. Endothelial cell injury induced by BPA through oxidative stress has been reported [30]. Insulin resistance is closely related to hypertension [31].

Effects of BPA binding to estrogen receptors on cardiovascular system might be different from those of estrogen. BPA has dual actions as an agonist and antagonist of estrogen for estrogen receptor alpha (ER*α*) but acts only as an agonist for estrogen receptor beta (ER*β*) [5]. Estrogen is important for maintaining and repairing endothelium mediated by ER*α* through increase in eNOS and cyclooxygenase resulting in vasorelaxation [20, 21]. The higher prevalence of hypertension among postmenopausal women might be attributed to the decline in estrogen level; however, it is not likely to affect the differences by menopausal status in the magnitude of association between BPA and hypertension. The effect of BPA as estrogen disruptor might be stronger in postmenopausal women when natural estrogen declines and is replaced with BPA. The significant association in postmenopausal but not in premenopausal women should be interpreted with caution. The greater and significant association in postmenopausal women might be due to the larger sample size in this age group. However, it is possible that this group of women had lower level of estrogen and the effect of BPA on estrogen receptor became dominant. The association was not related to hormone replacement, as the analysis with exclusion of women with history of taking hormone supplement did not change the results.

The different degrees of association according to criteria for hypertension were observed. The association was strongest for all hypertension but was weaker for high SBP and high DBP. This could be due to the fact that some individuals with hypertension were treated medically to lower BP level leading to an attenuated magnitude of association. For treated or untreated hypertension, the associations were relatively consistent suggesting that the medication for hypertension was not likely to affect the association.

There are some limitations in the present study. First, the present study used serum BPA as surrogate for BPA exposure which may have low sensitivity to reflect the burden of BPA in the body compared to urine BPA [32]. However, serum BPA reflected true level of BPA that is active [33]. Nonetheless, the underestimation of BPA levels might attenuate the association between BPA and hypertension. Second, the prevalence of hypertension (18.0%) in this population is not so high which might limit the power of the test for comparison among some subgroups and the nonsignificant findings in men or in premenopausal women might be due to the small sample size. It is possible that the association of BPA and hypertension might be confounded by other nutritional intakes such as salt intake as it is a risk factor for hypertension and it is possible that those who consumed a large amount of water or food contaminated with BPA also had high Na intake; however, the results were not likely to be confounded by common risk factors such as age, sex, alcohol intake, and diabetes status. The present study lacked data on medication used and was unable to look at the effect of antihypertension drugs and this issue needs further study. However, this study had strength as the sample was randomly selected as representative of general population.

In conclusion, the present study revealed that exposure to BPA was positively associated with hypertension and the results were not confounded by common risk factors. Effect of BPA on high blood pressure might be stronger in postmenopausal women; however, its mechanism related to estrogen on blood pressure needs further investigation.

## Figures and Tables

**Table 1:** Basic characteristics of the study samples.

	Men (*n* = 1275)	Women (*n* = 1283)	Total (*n* = 2558)	*P* value
Age, yr^a^	39.58 (16.95)	41.12 (16.64)	40.37 (16.81)	0.02
BPA, ng/mL^a^	0.35 (0.02)	0.33 (0.02)	0.34 (0.02)	0.22
SBP, mm Hg^a^	122.81 (16.01)	118.99 (17.46)	120.86 (16.89)	<0.001
DBP, mm Hg^a^	75.70 (10.93)	73.49 (10.23)	74.57 (10.63)	<0.001
Diabetes^b^	121 (5.01)	143 (6.17)	264 (5.60)	0.13
All HT^b^	440 (18.62)	422 (17.46)	862 (18.03)	0.51
Unaware HT^b^	204 (11.62)	152 (6.92)	356 (9.22)	0.001
HT with treatment^b^	195 (5.22)	235 (8.82)	430 (7.06)	<0.001
HT without treatment^b^	245 (13.39)	187 (8.63)	432 (10.98)	<0.01
Smoking^b^	416 (39.61)	46 (1.60)	462 (20.18)	<0.001
Alcohol drinking^b^	663 (67.96)	267 (25.46)	930 (46.27)	<0.001
BMI, kg/m^2^ ^a^	22.74 (3.99)	24.44 (4.87)	23.61 (4.55)	<0.001
BMI ≥ 25 kg/m^2^ ^b^	331 (24.05)	481 (40.75)	812 (32.59)	<0.001
E2, pg/mL^a^	36.83 (22.55)	70.08 (97.81)	53.79 (74.20)	<0.001

^a^Mean (SD),^ b^number (percentage).

BPA: bisphenol A, SBP: systolic blood pressure, DBP: diastolic blood pressure, HT: hypertension, BMI: body mass index, and E2: estradiol.

**Table 2:** Geographic mean (SE) of serum BPA by blood pressure status and selected factors among Thai men and women.

Condition	Geometric mean of BPA (ng/mL)								
	Men (*n* = 1275)			Women (*n* = 1283)			Total (*n* = 2558)		
	No	Yes	*P* value	No	Yes	*P* value	No	Yes	*P* value
All HT	0.34 (0.02)	0.43 (0.04)	0.05	0.30 (0.02)	0.47 (0.04)	<0.001	0.32 (0.02)	0.45 (0.03)	<0.001
Unaware HT	0.35 (0.02)	0.37 (0.06)	0.76	0.32 (0.02)	0.49 (0.05)	<0.001	0.34 (0.02)	0.41 (0.04)	0.07
SBP ≥ 140 mmHg	0.35 (0.02)	0.40 (0.06)	0.34	0.31 (0.02)	0.50 (0.04)	<0.001	0.33 (0.02)	0.45 (0.04)	<0.01
DBP ≥ 90 mmHg	0.35 (0.02)	0.43 (0.05)	0.14	0.32 (0.02)	0.43 (0.05)	0.05	0.34 (0.02)	0.43 (0.04)	0.04
HT with treatment	0.35 (0.02)	0.51 (0.05)	<0.01	0.32 (0.02)	0.49 (0.04)	<0.001	0.33 (0.01)	0.50 (0.03)	<0.001
BMI ≥ 25 kg/m^2^	0.35 (0.02)	0.36 (0.04)	0.05	0.33 (0.02)	0.33 (0.03)	0.82	0.34 (0.02)	0.34 (0.02)	0.83
Smoking	0.34 (0.02)	0.38 (0.03)	0.28	0.33 (0.02)	0.38 (0.07)	0.41	0.33 (0.02)	0.38 (0.03)	0.13
Alcohol drinking	0.43 (0.3)	0.32 (0.02)	<0.01	0.33 (0.02)	0.33 (0.03)	0.90	0.36 (0.02)	0.32 (0.02)	0.11
Menopause	—	—	—	0.32 (0.02)	0.36 (0.02)	0.09	—	—	—

BPA: bisphenol A, HT: hypertension, and BMI: body mass index.

**Table 3:** Age-adjusted mean (SE) and proportions (%) of selected characteristics in Thai population according to quartile of serum BPA in Thai population.

	All (*n* = 2558)					Men (*n* = 1275)					Women (*n* = 1283)				
	Q1	Q2	Q3	Q4	*P* for trends	Q1	Q2	Q3	Q4	*P* for trends	Q1	Q2	Q3	Q4	*P* for trends
All															
Age, yr^a^	38.83 (0.56)	38.62 (0.52)	40.82 (0.64)	44.52 (0.83)	<0.001	38.05 (0.72)	36.23 (0.67)	40.51 (0.84)	44.79 (1.01)	<0.001	39.47 (0.70)	41.07 (0.72)	41.09 (0.94)	44.20 (1.04)	<0.01
BMI, kg/m^2^ ^a^	23.66 (0.15)	23.80 (0.20)	23.56 (0.22)	23.34 (0.22)	0.24	22.64 (0.24)	22.89 (0.25)	23.13 (0.29)	22.35 (0.24)	0.68	24.49 (0.20)	24.68 (0.31)	23.94 (0.29)	24.53 (0.37)	0.57
SBP mmHg^a^	120.68 (0.54)	120.74 (0.43)	120.56 (0.51)	121.62 (0.79)	0.36	122.84 (0.84)	123.13 (0.61)	122.31 (0.62)	123.99 (1.08)	0.47	118.83 (0.74)	117.81 (0.68)	118.90 (0.79)	118.94 (1.17)	0.80
DBP mmHg^a^	74.58 (0.35)	74.56 (0.34)	74.33 (0.36)	74.83 (0.49)	0.83	75.11 (0.60)	75.94 (0.49)	75.64 (0.51)	76.75 (0.81)	0.13	74.17 (0.39)	73.18 (0.49)	73.14 (0.59)	72.54 (0.64)	0.04
Diabetes^b^	4.44 (0.79)	4.12 (0.78)	6.90 (0.93)	7.15 (0.88)	0.01	3.14 (0.68)	4.93 (1.54)	6.45 (1.37)	6.18 (1.02)	0.03	5.42 (1.12)	3.37 (0.53)	7.26 (1.13)	8.29 (1.34)	0.03
All HT^b^	15.44 (0.94)	17.59 (1.3)	16.63 (1.31)	23.22 (1.49)	0.001	18.11 (1.81)	18.31 (2.0)	17.18 (1.8)	23.11 (2.1)	0.07	13.3 (1.5 6)	16.77 (1.76)	16.00 (1.83)	23.32 (1.56)	<0.01
Unaware HT^b^	7.97 (0.77)	9.40 (1.15)	7.91 (1.03)	11.95 (1.38)	0.028	12.90 (1.82)	11.03 (2.07)	9.90 (1.44)	13.52 (1.87)	0.94	4.04 (1.04)	7.83 (1.04)	6.08 (1.24)	0.96 (2.01)	0.03
HT with treatment^b^	6.37 (0.70)	6.70 (0.87)	6.36 (0.80)	8.78 (0.77)	0.02	4.66 (1.01)	5.69 (1.10)	5.57 (1.19)	6.04 (0.95)	0.29	7.64 (1.0)	7.57 (1.20)	7.08 (1.04)	12.06 (1.30)	0.03
HT without treatment^b^	9.17 (0.72)	10.83 (1.11)	10.19 (1.00)	14.30 (1.51)	<0.001	13.49 (1.79)	12.53 (2.03)	11.60 (1.44)	17.05 (2.07)	0.25	5.75 (1.22)	9.21 (1.29)	8.87 (1.45)	10.98 (2.12)	0.04
SBP ≥ 140 mmHg^b^	10.68 (0.86)	12.37 (0.95)	9.52 (0.89)	15.12 (1.62)	0.06	13.4 (1.63)	13.65 (1.70)	9.11 (1.44)	16.08 (1.98)	0.60	8.49 (1.49)	11.10 (1.34)	9.79 (1.32)	13.94 (2.35)	0.05
DBP ≥ 90 mmHg^b^	7.73 (1.00)	7.99 (0.98)	8.42 (1.13)	9.24 (1.17)	0.36	11.92 (1.72)	9.08 (1.39)	8.14 (1.36)	11.86 (1.94)	0.88	4.46 (1.02)	7.09 (1.33)	8.69 (1.67)	6.07 (1.40)	0.15
Smoking^b^	20.00 (1.56)	20.83 (1.99)	16.86 (1.58)	23.37 (2.28)	0.62	43.05 (2.89)	39.89 (3.09)	33.41 (2.54)	41.70 (3.69)	0.43	1.45 (0.55)	1.67 (0.43)	1.73 (0.67)	1.33 (0.43)	0.92
Alcohol drinking^b^	6.75 (0.70)	12.09 (1.98)	6.88 (1.03)	7.64 (1.16)	0.79	14.21 (1.58)	21.05 (3.25)	13.58 (1.89)	13.62 (2.16)	0.35	0.57 (0.17)	2.42 (0.68)	0.60 (0.25)	0.28 (0.17)	0.10
E2, (pg/mL)^a^	56.51 (3.64)	48.02 (2.37)	57.32 (3.66)	53.41 (2.91)	0.92	39.66 (2.81)	35.08 (0.63)	35.08 (0.74)	37.52 (0.90)	0.38	71 (5.69)	65.73 (4.85)	78.52 (5.81)	70.67 (5.91)	0.60

^a^Mean (SE) and^ b^percentage (SE).

BPA: bisphenol A, BMI: body mass index, SBP: systolic blood pressure, DBP: diastolic blood pressure, HT: hypertension, E2: estradiol, and Q: quartile.

**Table 4:** Adjusted odds ratios for high blood pressure associated with log⁡(BPA) and E2 level with interaction term of log⁡(BPA) and E2 level among women.

Variables	All HT	*P* value	Unaware HT	*P* value	SBP ≥ 140 mm Hg	*P* value
Age	1.07 (1.05, 1.08)	<0.001	1.04 (1.03, 1.05)	<0.001	1.07 (1.05, 1.08)	<0.001
BMI	1.10 (1.06, 1.14)	<0.001	1.08 (1.04, 1.13)	<0.001	1.10 (1.07, 1.14)	<0.001
Diabetes	2.22 (1.46, 3.39)	0.001	0.81 (0.46, 1.42)	0.43	1.75 (1.11, 2.77)	0.02
Smoking	1.38 (0.62, 3.08)	0.41	1.20 (0.43, 3.31)	0.71	0.71 (0.27, 1.84)	0.46
log⁡(BPA)	1.09 (1.02, 1.16)	0.01	1.11 (1.00, 1.22)	0.06	1.04 (0.95, 1.13)	0.42
E2	0.99 (0.99, 1.00)	0.037	0.99 (0.99, 1.00)	0.05	0.99 (0.99, 1.00)	0.19
log⁡(BPA) × E2	1.0 (1.0, 1.0)	0.20	1.0 (1.0, 1.00)	0.79	1.00 (0.99, 1.00)	0.68

BPA: bisphenol A, BMI: body mass index, HT: hypertension, SBP: systolic blood pressure, and E2: estradiol.

**Table 5:** Adjusted odds ratios for high blood pressure according to quartile of serum BPA.

	Odds ratios (95% CI)			
	Q1	Q2	Q3	Q4
All				
All HT	1	1.15 (0.92, 1.44)	1.08 (0.79, 1.47)	1.76 (1.33, 2.33)
Unaware HT	1	1.13 (0.78, 1.66)	0.97 (0.65, 1.45)	1.49 (1.05, 2.12)
SBP ≥ 140 mm Hg	1	1.14 (0.84, 1.54)	0.84 (0.59, 1.19)	1.52 (1.05, 2.21)
DBP ≥ 90 mm Hg	1	0.94 (0.64, 1.36)	1.09 (0.70, 1.70)	1.17 (0.74, 1.84)
HT with treatment	1	1.09 (0.69, 1.71)	0.95 (0.62, 1.47)	1.48 (1.07, 2.04)
HT without treatment	1	1.13 (0.84, 1.52)	1.11 (0.80, 1.53)	1.62 (1.16, 2.25)
Men				
All HT	1	0.93 (0.59, 1.45)	0.82 (0.51, 1.32)	1.44 (0.99, 2.09)
Unaware HT	1	0.79 (0.43, 1.46)	0.71 (0.43, 1.17)	1.09 (0.69, 1.71)
SBP ≥ 140 mm Hg	1	0.93 (0.53, 1.63)	0.57 (0.34, 0.94)	1.30 (0.83, 2.06)
DBP ≥ 90 mm Hg	1	0.65 (0.41, 1.04)	0.61 (0.34, 1.09)	1.04 (0.59, 1.84)
HT with treatment	1	1.28 (0.65, 2.52)	1.07 (0.47, 2.42)	1.26 (0.71, 2.21)
HT without treatment	1	0.84 (0.49, 1.47)	0.79 (0.49, 1.28)	1.39 (0.91, 2.12)
Women				
All HT	1	1.41 (0.92, 2.15)	1.35 (0.80, 2.28)	2.16 (1.31, 3.56)
Unaware HT	1	2.03 (1.05, 3.94)	1.61 (0.74, 3.49)	2.54 (1.11, 6.83)
SBP ≥ 140 mm Hg	1	1.41 (0.81, 2.46)	1.21 (0.71, 2.05)	1.81 (0.96, 3.42)
DBP ≥ 90 mm Hg	1	1.61 (1.13, 4.46)	2.24 (1.13, 4.46)	1.28 (0.60, 2.71)
HT with treatment	1	1.01 (0.58, 1.73)	0.86 (0.48, 1.52)	1.65 (1.05, 2.58)
HT without treatment	1	1.66 (0.93, 2.97)	1.71 (0.92, 3.16)	1.99 (0.94, 4.19)

Model adjusted for sex, age, BMI, diabetes, smoking, and alcohol drink.

BPA: bisphenol A, HT: hypertension, SBP: systolic blood pressure, DBP: diastolic blood pressure, and BMI: body mass index.

**Table 6:** Adjusted odds ratio (AOR) for high blood pressure according to quartile of serum BPA among premenopausal and postmenopausal women.

	Odds ratios (95% CI)			
	BPA Q1	BPA Q2	BPA Q3	BPA Q4
Premenopause (*n* = 512)				
All HT	1	1.43 (0.69, 2.94)	1.55 (0.55, 4.34)	2.12 (0.87, 5.19)
Unaware HT	1	1.54 (0.54, 4.41)	1.96 (0.53, 7.31)	3.01 (0.81, 11.22)
SBP ≥ 140 mm Hg	1	1.72 (0.70, 4.20)	2.11 (0.68, 6.52)	2.75 (0.93, 8.10)
DBP ≥ 90 mm Hg	1	1.59 (0.57, 4.45)	2.94 (1.11, 7.8)	1.03 (0.32, 3.35)
HT with treatment	1	2.34 (0.73, 7.48)	0.15 (0.02, 1.35)	1.66 (0.59, 4.71)
HT without treatment	1	1.02 (0.40, 2.61)	2.25 (0.86, 5.91)	1.98 (0.59, 6.58)
Postmenopause (*n* = 771)				
All HT	1	1.56 (0.94, 2.57)	1.44 (0.88, 2.37)	2.33 (1.31, 4.15)
Unaware HT	1	2.94 (1.42, 6.07)	1.80 (0.91, 3.59)	2.47 (0.92, 6.58)
SBP ≥ 140 mm Hg	1	1.43 (0.78, 2.62)	1.10 (0.62, 1.95)	1.47 (0.73, 2.94)
DBP ≥ 90 mm Hg	1	2.05 (0.78, 5.38)	2.47 (0.87, 7.01)	1.92 (0.53, 6.99)
HT with treatment	1	0.77 (0.44, 1.36)	1.18 (0.64, 2.17)	1.74 (0.99, 3.07)
HT without treatment	1	3.02 (1.55, 5.90)	1.69 (0.90, 3.18)	2.26 (0.95, 5.42)

Postmenopause status was defined as self-reported of no menstruation or women aged ≥ 60 years.

BPA: bisphenol A, HT: hypertension, SBP: systolic blood pressure, and DBP: diastolic blood pressure.

**Table 7:** Adjusted odds ratio (AOR) for high blood pressure associated with log⁡(BPA) in premenopausal and postmenopausal women.

	Premenopausal (*n* = 512)	Postmenopausal (*n* = 771)
All HT	1.06 (0.97, 1.17)	1.08 (1.01, 1.06)
Unaware HT	1.07 (0.92, 1.23)	1.15 (1.03, 1.28)
SBP ≥ 140 mm Hg	1.07 (0.95, 1.21)	1.04 (0.96, 1.13)
DBP ≥ 90 mm Hg	1.07 (0.96, 1.20)	1.10 (0.93, 1.29)
HT with treatment	1.00 (0.88, 1.15)	1.02 (0.94, 1.11)
HT without treatment	1.07 (0.96, 1.20)	1.13 (1.03, 1.25)

Model adjusted for age, BMI, diabetes, smoking, and E2.

BPA: bisphenol A, HT: hypertension, and E2: estradiol.
